# Diverse urban plantings managed with sufficient resource availability can increase plant productivity and arthropod diversity

**DOI:** 10.3389/fpls.2014.00517

**Published:** 2014-10-30

**Authors:** Jonathon N. Muller, Susan Loh, Ligia Braggion, Stephen Cameron, Jennifer L. Firn

**Affiliations:** ^1^School of Earth, Environmental and Biological Sciences, Queensland University of TechnologyBrisbane, QLD, Australia; ^2^Creative Industries, School of Design, Queensland University of TechnologyBrisbane, QLD, Australia; ^3^Department of Forest Sciences, Agricultural Sciences Faculty, São Paulo State UniversityBotucatu, Brazil

**Keywords:** urban biodiversity, ecosystem functions, ecosystem services, plant diversity, arthropod diversity, plant CO_2_

## Abstract

Buildings structures and surfaces are explicitly being used to grow plants, and these “urban plantings” are generally designed for aesthetic value. Urban plantings also have the potential to contribute significant “ecological values” by increasing urban habitat for animals such as arthropods and by increasing plant productivity. In this study, we evaluated how the provision of these additional ecological values is affected by plant species richness; the availability of essential resources for plants, such as water, light, space; and soil characteristics. We sampled 33 plantings located on the exterior of three buildings in the urban center of Brisbane, Australia (subtropical climatic region) over 2, 6 week sampling periods characterized by different temperature and rainfall conditions. Plant cover was estimated as a surrogate for productivity as destructive sampling of biomass was not possible. We measured weekly light levels (photosynthetically active radiation), plant CO_2_ assimilation, soil CO_2_ efflux, and arthropod diversity. Differences in plant cover were best explained by a three-way interaction of plant species richness, management water regime and sampling period. As the richness of plant species increased in a planter, productivity and total arthropod richness also increased significantly—likely due to greater habitat heterogeneity and quality. Overall we found urban plantings can provide additional ecological values if essential resources are maintained within a planter such as water, light and soil temperature. Diverse urban plantings that are managed with these principles in mind can contribute to the attraction of diverse arthropod communities, and lead to increased plant productivity within a dense urban context.

## Introduction

Rapid human concentration in cities, predicted to increase to 70% by 2030 (Unfpa, 2011), have led to great changes to ecosystems that erode biodiversity that in turn alters ecological processes vital to human well-being (Millennium Ecosystem Assessment, 2005). Ecosystem services are the benefits humans derive from ecosystem functions, e.g., carbon dioxide (CO_2_) gas exchange and nutrient cycling that drive greenhouse gas regulation via plant growth services (Chapin et al., 1996). Studies have found that ecosystem services are regulated by both the diversity (Naeem et al., 1995; Tilman et al., 1996) and identity (Hooper et al., 2005) of the plant and animal species living in a community, making it vital to address the issue of declining biodiversity in our cities (Kendal et al., 2012). Climate change impacts and loss of habitat for biodiversity are two key challenges that could be mitigated through better management of urban biodiversity (Davies et al., 2011).

Despite clear evidence that bio-diverse and healthy ecosystems are beneficial to human health and wellbeing (Costanza et al., 1997), it is only recently that biodiversity has been considered when designing buildings (Daily, 1997). Roofs and walls of buildings can be used to grow plants including traditionally styled planter boxes designed and built integrally into the building structure. These “urban plantings” (see Box 1 for generic definitions) represent ecologically underutilized space that could be transformed into green space (Dunnett and Kingsbury, 2004). Previous studies have focused on infrastructure and engineering related benefits that urban plantings can provide such as temperature reduction (Alexandri and Jones, 2008) and stormwater runoff reduction (Getter et al., 2007), but few studies have examined the use of urban plantings to mitigate climate change through CO_2_ sequestration and to provide refuge habitat for biodiversity (Hooper and Vitousek, 1998; Oberndorfer et al., 2007; Cook-Patton et al., 2011). To address these two key knowledge gaps, our study evaluates how the plants, soils and habitat provision of urban plantings change with plant species richness and resource availability.

Box 1 **Definitions of key terms and concepts.**

**Urban plantings** describe any type of vegetated building surface or structure, such as green roofs, green walls, green facades using raised planter and trellis system and also traditionally styled planter boxes designed and built integrally into a building's structure.

**Green roof** refers to any horizontal building space such as a rooftop or podium that has been partially or completely covered in several layers including waterproofing, drainage, soil substrate and vegetation. **Intensive green roofs** are essentially rooftop gardens, with greater than 150 mm substrate depth and require high maintenance. They are usually accessible, and are designed for aesthetic or recreational purposes, much like a regular garden. Intensive green roofs usually need to be incorporated into the building design, due to the weight bearing issues of substrate and vegetation. **Extensive green roofs** consist of shallow substrates of 50–150 mm depth and require little to no maintenance. They are usually inaccessible as they are primarily designed to provide environmental benefits. Extensive green roofs are relatively light-weight therefore they can usually be retrofitted to existing building rooftops.

**Ecosystem functions** are the physical, chemical, and biological processes or attributes that contribute to the self-maintenance of an ecosystem; in other words, what the ecosystem does.

**Ecosystem services** are the beneficial outcomes humans derive from ecosystem functions.

**Diversity** is a general term that can be defined at multiple levels and encompasses variation within and among species.

**Richness** refers to the number of species or genotype present in an assemblage, but does not describe the differences among these units.

A substantial proportion of the CO_2_ emissions produced in cities originates from buildings (Newman, 2006), yet buildings can also help reduce atmospheric CO_2_ by incorporating plants and soils into their design via urban plantings. Urban plantings are highly managed systems, and generally have controlled and known abiotic factors such as water, soil composition, and age. Plant diversity may have a significant influence on productivity, and subsequently CO_2_ uptake (Davies et al., 2011) and greater plant species diversity has been shown to produce greater carbon sequestration in grassland experiments (Tilman et al., 2006). Urban plantings have similar restricted growing conditions to green roofs. A recent study in Michigan (Whittinghill et al., 2014) examined carbon sequestration of three different types of green roofs of varying complexity and suggested that plant biomass and more complex plant communities increase the amount of carbon sequestered. However, to date, no green roof or urban planter study has specifically examined and tested the influence of plant diversity on carbon sequestration.

Urban plantings can also provide habitat and food for a variety of organisms and contribute to increased biodiversity in cities. Urban plantings may have greater importance as long-term habitats for smaller organisms such as arthropods due to the loss of their original habitats—many of these arthropods require specific micro-habitats to maintain viable populations (Gaston et al., 1998). Arthropods sampled in urban plantings have been shown to improve ecosystem function by contributing to soil forming processes (Schrader and Böning, 2006; Rumble and Gange, 2013), controlling pest insects (Hunter and Hunter, 2008), and pollinating plants (Hunter, 2002; McKinney, 2008; Colla et al., 2009). A study by Schindler et al. (2011) using pitfall traps found arthropod species richness to increase with increased vegetation cover on green roofs. Similarly, a recent study by Madre et al. (2013) found a positive relationship between arthropod species richness and plant species richness, using a standardized hand sampling method on the ground level, and within vegetation layers.

Increased density of cities means open green spaces are sometimes substituted with small plantings that are incorporated into a building. In this study, we measure plant diversity in relationship to how leaf CO_2_ assimilation, soil CO_2_ respiration, and arthropod diversity vary with plant species richness and resource availability across 33 plantings positioned around the exteriors of three buildings that are found within a 3 km radius of the CBD of Brisbane to address the following questions:

1. Does either plant cover (a surrogate for productivity) vary with plant species richness, planter position and size, and resource availability (i.e., light and water)?
2. Does plant CO_2_ assimilation vary with plant richness, planter position and size, and resource availability (i.e., light and water)?
3. Does soil CO_2_ efflux vary with plant species richness, planter position and size, and resource availability (i.e., light and water)?
4. Does arthropod diversity in urban plantings vary with plant species richness, planter position and size, and resource availability (i.e., light and water)?
5. How does motility and accessibility influence the arthropod diversity found?

Measurements showing positive results from our initial queries above would indicate that urban plantings can support healthy biodiversity under managed microclimatic conditions, and therefore could provide additional ecological values in a dense urban environment.

## Materials and methods

### Site description

**Figure 1:**
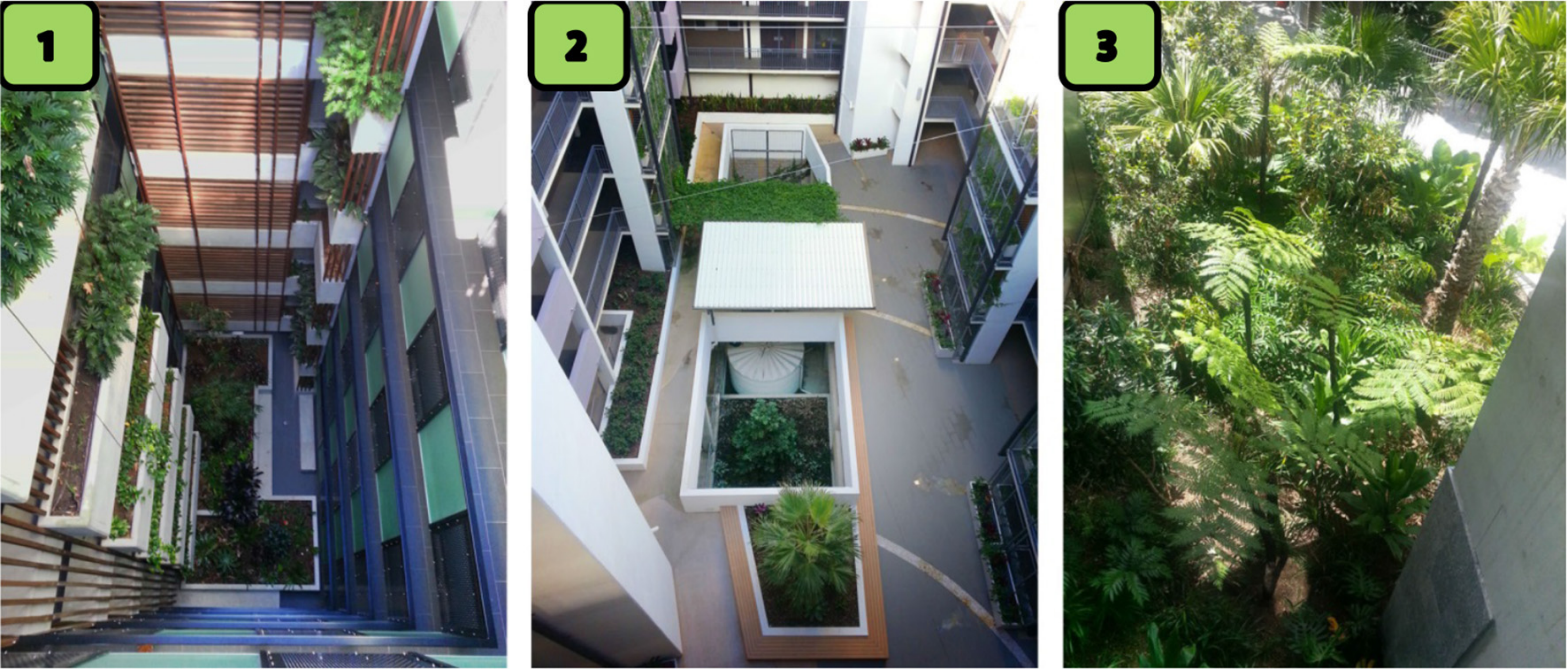
**Photographs of the three building sites, labeled as 1–3 used in this study to show that all plantings were outside the building**.

This study was conducted on three building sites in Brisbane, Queensland (Figure 1). Building three was situated closest to the city center (1 km), followed by building one (1.9 km) and building two (2.7 km). Two periods of sampling (hereafter referred to as sampling times) were performed between July and September 2013, over two seasons; winter and spring. The winter sampling time ran for 7 weeks, and the spring sampling period for 6 weeks. Building sites consisted of between 6 and 55 individual concrete planters built integrally within the building itself ranging in size from 1.5 × 0.35 × 0.4 m to 8.0 × 8.0 × 1.0 m (length × width × depth), which were all exposed to outside elements, i.e., light and rainfall, and were also managed with additional automatic drip-fed watering systems. A range of planters in each building were sampled to ensure that plantings with diverse characteristics were included in the study, for example light availability, distance from ground level, and area. A summary of sites and planter characteristics is available in Table 1, and specific sampling information for each site and sub-site, is available in Table S1 in the supplementary material. Site building plans of each site were used to determine planter area, depth, and distance from ground level. Information regarding building age, watering regime (measured as liters per 1 m^2^ per week), and soil properties was obtained directly from site managers. Seasonal variations of cloud cover (eighths), humidity (%), precipitation (mm), and air temperature (°C) were recorded for each day of sampling, as these climactic variables may impact plant, and arthropod communities (Kremen et al., 1993; Geider et al., 2001). Climate data for the area encompassing the three study sites was sourced from the Bureau of Meteorology Brisbane weather station (Australian Government, 2013).

**Table 1:** **Summary of sites and planter characteristics including number of plantings sampled number sampled (total number present), soil properties, age, water regime, and soil depth information was obtained from interviews with building managers**.

**Characteristic**	**Building one**	**Building two**	**Building three**
Number of plantings	12 (49)	15 (56)	6 (7)
Soil properties	Low organic content, loam and sand with hoop pine mulch	Low organic content, loam and sand with hoop pine mulch	Low organic content, loam and sand with tea tree mulch
Application of mulch	Once during initial planting phase	Once during initial planting phase	Once during initial planting phase
Watering regime (l/m^2^/week: Mean ± Standard deviation)	15.33 ± 0.44	15.71 ± 0.37	71.4 ± 0
Age (years)	4	3	1
Soil depth range (cm)	40–75	40–75	100
Area range (m^2^)	1–15	1–85	40–128
Distance to ground level range (m)	6.96–24.99	0–16.56	8–16
Distance to nearest green space (m)	50	25	40
Planter % vegetation cover (Mean ± Standard deviation)	59.8 ± 27.4	69 ± 19.8	143.5 ± 27.7
Plant richness (Mean ± Standard deviation)	4.1 ± 3.4	5.0 ± 0.9	10.5 ± 2.3

Throughout the 3 months of these experiments, sampling was always conducted between 9.00 am and 12.00 pm because these were the times when climatic conditions are most suitable for plants to be photosynthesizing. This is also a measure of control to compare processes across sampling times.

### Plant cover and plant CO_2_ assimilation (surrogates for productivity) measurements

Plant cover was estimated visually as percent cover for each planter using a modified Daubenmire method (Daubenmire, 1959) This method was used because it involves predicting the cover of all species within a plot, thus giving us a possibility of multiple layers of vegetation and therefore, values of cover above 100%. Plant cover is a commonly used estimate measure of plant primary productivity when destructive sampling is not possible (Röttgermann et al., 2000), and all plants were identified to species level (see Table S2 in the supplementary information for a detailed list of the plant species recorded).

Plant photosynthetic flux (μmol m^−2^ s^−1^) was measured as a proxy of plant CO_2_ assimilation rate (Hesketh and Baker, 1967). Leaf measurements were made *in situ* with an LI-6400 portable photosynthesis system, fitted with a leaf-chamber infrared gas analyser (LI-COR Inc.). Light information was measured with each individual leaf sample using the leaf-chamber's built-in light sensor. This sensor measured photosynthetically active radiation (hereafter PAR), a term which denotes the range of light wavelengths that can be used by green plants to photosynthesize. This method was used to gain a realistic indication of the rate of photosynthesis occurring within each of the sampled plantings. Individual plant leaves used in these measurements were tested at a concentration of 400 ppm CO_2_, as this is the average ambient CO_2_ level. CO_2_ flux measurements were then logged when photosynthetic activity stabilized. These measurements were taken on two plant leaves from randomly selected individual plants from the two dominant understory, and two dominant mid-story plant species of each planter. In plantings with fewer than four plant species present, measurements were made on the next highest number present. The same individual plants were sampled for both sampling times. Leaves from the dominant top-story plants were inaccessible and therefore, not included in this study for three reasons: (1) sampling was a health and safety risk, (2) they were absent from the majority of plantings (81.82%), and (3) even when present they provided substantially less total cover (7.8%) in comparison to the combined midstorey and understorey plants. For more information regarding plant photosynthetic sampling, refer to Table S1.

### Soil CO_2_ efflux measurements

Soil CO_2_ efflux (μmol m^−2^ s^−1^) was measured as a proxy of soil CO_2_ respiration rate (Donelan and Drennan, 1995). Measurements were made with an LI-6400 portable photosynthesis system (LI-COR Inc.), fitted with a 6400-09-soil CO_2_ flux chamber and temperature probe. Ambient CO_2_ concentration at the soil surface was recorded with the soil CO_2_ flux chamber. A probe was used to record soil temperature. The soil chamber was then inserted to a depth of 2 cm, where CO_2_ efflux based on the ambient concentration and soil temperature was computed. This measurement was cycled automatically for three iterations, and the final measurement logged. The average soil CO_2_ flux value based on these measurements was then calculated. These measurements were taken in each planting at randomly selected points every 10 m^2^. One single measurement was taken for individual plantings under 10 m^2^.

### Arthropod diversity measurements

To account for the presence of a wide range of arthropods across the different areas they inhabit, and also to assess arthropod dispersal, three different sampling methods were used: soil sampling, flight intercept traps, and direct sampling from plants. Morphospecies, which are species distinguished from others based on morphology, was used as a surrogate for identifying arthropods (Oliver and Beattie, 1996). To assess differing dispersal capabilities, arthropods were categorized as either winged or wingless. For a summary of arthropod species richness and abundance see Table S3.

Soil core samples were taken from each planter to provide an indication of soil dwelling arthropods. The soil core used in this study had a 54 mm diameter, and a depth of 100 mm. One sample was taken at a randomly selected point per 10 m^2^ of each planter. One single sample was taken for individual plantings under 10 m^2^. Arthropods in these soil samples were extracted with Tullgren funnels for 5 days (MacFadyen, 1953), with a 4 mm sieve (Upton, 1991). Specimens were preserved in propylene glycol before being sorted to morphospecies using a dissecting microscope at 100x magnification.

Sticky aphid/whitefly traps from Seabright Laboratories (Seabright Laboratories, 2013) were used to provide an indication of flying arthropods in each planter. Each trap used in this study was 16 × 10.2 cm in area, and reverse folded to expose an adhesive surface of bright yellow coloration. This specific hue of bright yellow is design to attract a wide-range of pest insects (Seabright Laboratories, 2013). Each trap was attached to a bamboo frame and suspended approximately 15 cm from the ground. The assembled traps were placed in each planter at randomly selected points every 10 m^2^. One single trap was placed in individual plantings under 10 m^2^. Sticky traps were present for the first 2 weeks of each sampling time, before being collected, and absent for the remainder of the sampling time. Collected sticky traps were stored in a fridge until sorted to morphospecies using a dissecting microscope at 100x magnification.

Standardized visual inspection was used to provide an indication of plant-dwelling arthropods (Gotelli et al., in press). Observations were made on selected individual plants, from each of the two dominant mid-story and two dominant understory plant species in each planter. We standardized this process by spending 10 min on each plant. Arthropods were provisionally identified to morphospecies on-site using a 10x hand lens, or were preserved in propylene glycol before being identified in the lab using a dissecting microscope at 100x magnification.

### Data analyses

We used linear mixed effects models (hereafter LMEMs) to analyse the effects of different abiotic and biotic variables on plant cover, plant CO_2_ assimilation rates, soil CO_2_ respiration rates and arthropod diversity. Models were set-up with random effects of planter nested within building and the fixed effects tested were plant species richness, water regime, establishment age, and planter size. The base unit of measurement used was individual plantings nested within each site. We used diagnostic plots to check model assumptions (Pinheiro and Bates, 2000). There was no evidence of correlation of observations within groups in any of the models so we assumed that within groups, errors were normally distributed. Finally, we used Wald tests to assess the significance of terms in the fixed effects part of the models (Pinheiro and Bates, 2000). We changed the order of fixed effects in the model structure to check if order affected the significance of relationships and we found no effect. The statistical program R version 3.0.2 (R Development Core Team, 2012) and the package nlme (nonlinear mixed-effects) were used for these analyses.

## Results

### Temperature and rainfall differences between sampling periods

**Figure 2:**
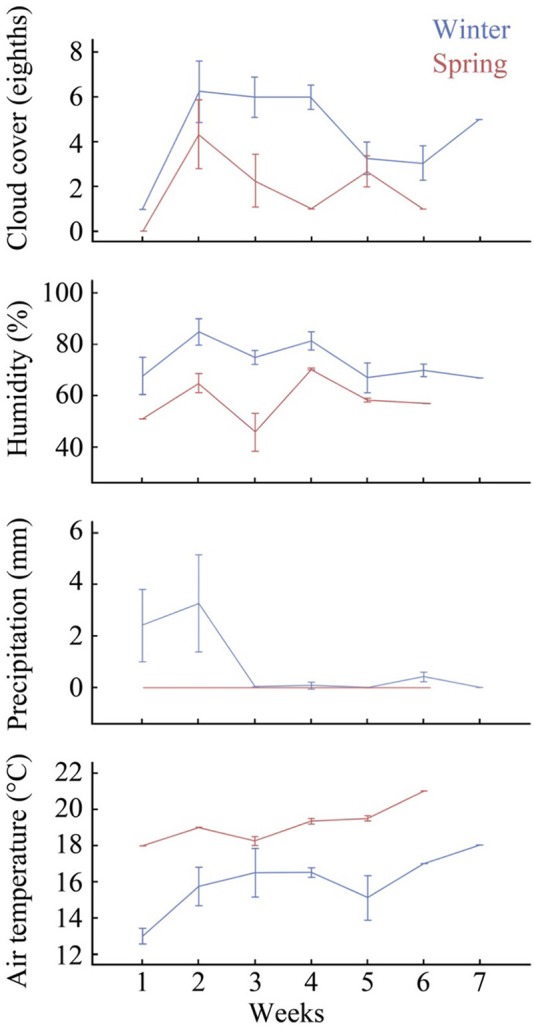
**Summary of climatic variables showing weekly average cloud cover (eighths), humidity (%), precipitation (mm), and air temperature (°C)**. Error bars indicate 95% confidence intervals, for winter and spring sampling times (7 and 6 weeks respectively). Data sourced from the Bureau of Meteorology Brisbane weather station (Australian Government, 2013).

On average, the winter sampling period had higher humidity, precipitation and cloud cover, and lower temperatures than the spring sampling period for each week of the study (Figure 2). Significant differences were found between the two seasons for cloud cover [*F*_(1, 28)_ = 6.15, *p* = 0.02], humidity [*F*_(1, 28)_ = 10.41, *p* = < 0.01], and air temperature [*F*_(1, 28)_ = 14.58, *p* = < 0.01], although the difference between season and precipitation were not significant [*F*_(1, 28)_ = 2.07, *p* = 0.16].

### Relationship between plant cover, plant richness, and resource availability in urban plantings

**Figure 3:**
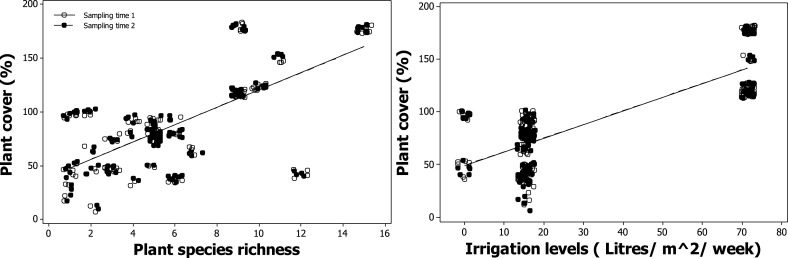
**Modeled relationship between plant cover (%), plant species richness and irrigation levels depending on the sampling time period**.

Plant species richness, management watering regimes and sampling time were found to be significant predictors of variation in plant cover in a three way interaction [*F*_(1, 227)_ = 18.09, *p* < 1 × 10^−4^; Table 2]. A positive relationship between plant species richness and plant cover was found [*F*_(1, 29)_ = 37.99, *p* = 0.01; Figure 3]. Plant cover varied marginally between winter and spring (Figure 3) because of the subtropical climate of the study sites. A positive relationship between irrigation and plant cover was also found [*F*_(1, 29)_ = 40.67,*p* < 1 × 10^−4^; Figure 3]. PAR, CO_2_ assimilation rate, soil depth, area or establishment age were not significantly correlated with plant cover.

**Table 2:** **Results from a Wald test of a linear mixed effect model with the response variable plant cover conducted to assess the significance of the fixed effects (i.e., PAR, CO_2_ assimilation rate, sampling times, watering regime, soil depth, planter area, and establishment age)**.

**Variables**	**numDF**	**denDF**	***F*-value**	***p*-value**
Plant species richness	1	29	37.99	0.01
Establishment age	1	1	19.60	0.14
Area	1	1	27.98	0.12
Soil depth	1	29	0.08	0.78
Watering regime	1	29	40.67	<1 × 10^−4^
Sampling time	1	230	176.87	<1 × 10^−4^
CO_2_ assimilation rate	1	230	0.71	0.40
PAR	1	230	1.24	0.27
Plant species richness: watering regime	1	27	0.73	0.40
Plant species richness: sampling time	1	227	382.98	<1 × 10^−4^
Watering regime: sampling time	1	227	146.96	<1 × 10^−4^
Plant species richness: watering regime: sampling time	1	227	18.09	<1 × 10^−4^

Plant species richness did not vary from winter to spring and ranged between 1 and 15 plant species per planter, with building three having the highest average (10.5 ± 2.3), followed by building two (5.0 ± 0.9), and building one (4.1 ± 3.4). Building three was found to have the overall highest plant cover for both winter and spring (143.5 ± 27.7 and 152 ± 25.4; Figure S1) compared to building two (69 ± 19.8 and 70.4 ± 20.4; Figure S1) and building one (59.8 ± 27.4 and 60.2 ± 27.7; Figure S1). Building three received five times the amount of irrigation (71.4 l/m^2^/week, Table 1).

### Relationship between plant CO_2_ assimilation rate, plant richness, and resource availability in urban plantings

**Figure 4:**
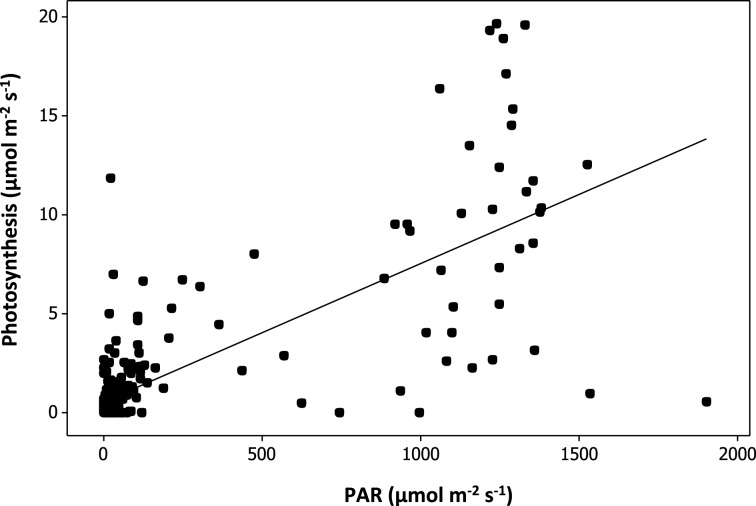
**Modeled relationship between plant CO_2_ assimilation rates and photosynthetically active radiation (PAR) measurement taken from above of the leaves measured**.

The relationship between plant species richness and plant CO_2_ assimilation rate (Figure 4) was not significant [*F*_(1, 29)_ = 0.64, *p* = 0.43; Table 3]. PAR was found to have a strong significant positive relationship with plant CO_2_ assimilation rates [*F*_(1, 230)_ = 297.17, *p* < 1 × 10^−4^; Table 3, Figure 4], where increased light correlated with increased plant CO_2_ assimilation rates. Variation in plant CO_2_ assimilation rates was not explained by sampling time, watering regime, soil depth, area, establishment age, or plant cover (Table 3).

**Table 3:** **Results from a Wald test of a linear mixed effect model with the response variable assimilation rate to assess the significance of the fixed effects (i.e., PAR, sampling times, watering regime, soil depth, area, establishment age, plant species richness, and plant cover) on a linear mixed effects model for plant CO_2_ assimilation rate**.

**Variables**	**numDF**	**denDF**	***F*-value**	***p*-value**
Plant cover	1	230	1.49	0.23
Plant species richness	1	29	0.64	0.43
Establishment age	1	1	5.30	0.26
Area	1	1	15.71	0.16
Soil depth	1	29	0.38	0.54
Watering regime	1	29	1.31	0.26
Sampling time	1	230	1.19	0.28
PAR	1	230	296.17	<1 × 10^−4^

### Relationship between soil CO_2_ efflux, plant richness, and resource availability in urban plantings

**Figure 5:**
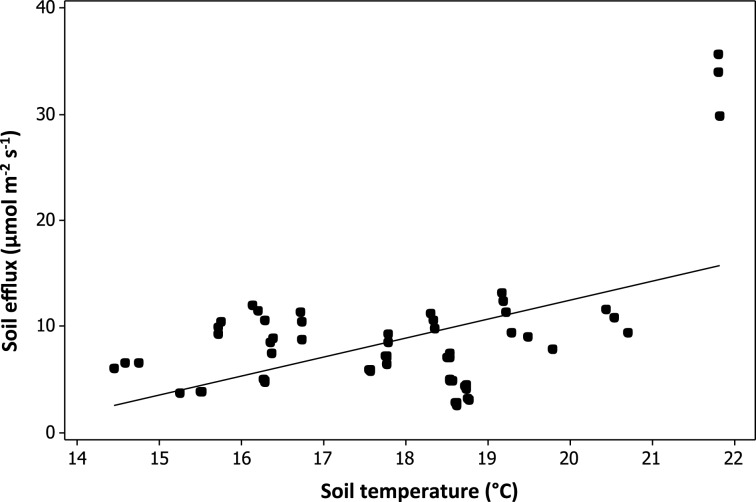
**Modeled relationship between soil CO_2_ respiration and soil temperature**.

No significant relationship was found between plant species richness and soil CO_2_ respiration [*F*_(1, 29)_ = 0.38, *p* = 0.54; Table 4]. Soil temperature was found to have a significant positive relationship with soil CO_2_ respiration [*F*_(1, 235_ = 217.48, *p* < 0.01; Table 4, Figure 5]. Variation in soil CO_2_ respiration rates could not be explained by sampling times, establishment age, area, soil depth, or watering regime (Table 4).

**Table 4:** **Results from a Wald test of a linear mixed effect model with the response variable soil CO_2_ respiration to assess the significance of the fixed effects (i.e., PAR, sampling times, water regime, planter depth, planter area, establishment age, soil arthropod species richness, soil arthropod species abundance, plant species richness, and plant coverage) on a linear mixed effects model for soil CO_2_ respiration**.

**Variables**	**numDF**	**denDF**	***F*-value**	***p*-value**
Plant cover	1	29	0.39	0.54
Plant species richness	1	29	0.38	0.54
Age	1	1	0.29	0.69
Area	1	30	0.06	0.80
Soil depth	1	29	0.09	0.76
Watering regime	1	29	1.12	0.30
Sampling times	1	235	1.44	0.23
Soil temperature	1	235	217.48	<1 × 10^−4^

### Relationship between total arthropod richness, plant richness, and resource availability in urban plantings

**Figure 6:**
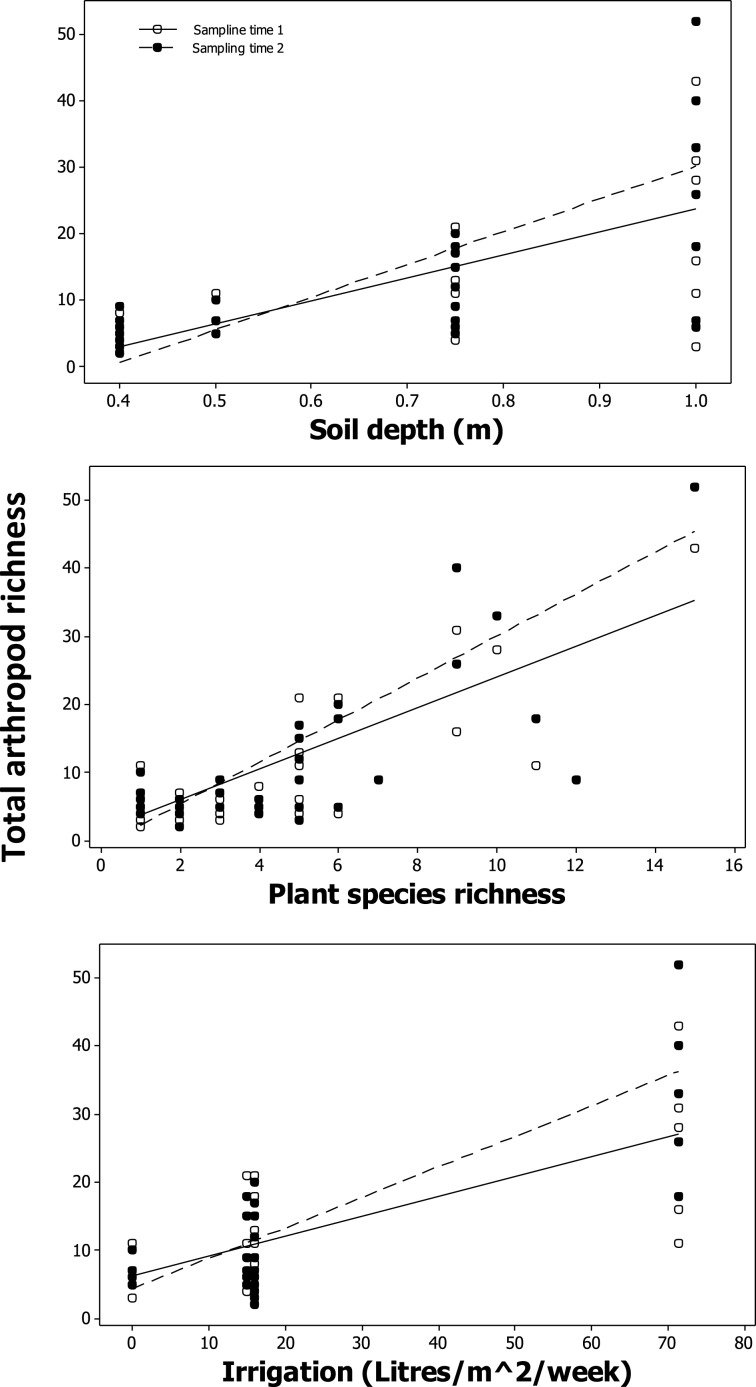
**Modeled relationship between total arthropod richness, soil depth, plant species richness, and irrigation levels**.

A significant positive relationship was found between total arthropod species richness and plant species richness [*F*_(1, 29)_ = 6.04, *p* = 0.02; Table 5, Figure 6], soil depth [*F*_(1, 29)_ = 11.71, *p* < 1.9 × 10^−3^; Table 5, Figure 6], and watering regime [*F*_(1, 29)_ = 27.64, *p* < 1 × 10^−4^; Table 5, Figure 6]. Total arthropod richness was higher during second “spring” sampling time period [*F*_(1, 230)_ = 69.02, *p* = < 1 × 10^−4^; Table 5; Figure 6], with plant species richness, soil depth, and watering regime each showing a significant two-way interaction with sampling time (Table 5). Variation in total arthropod species richness was not explained by establishment age, area of the planter, distance to the ground, or distance to the nearest green space (Table 5).

**Table 5:** **Results from a Wald test of a linear mixed effect model with the response variable total species richness to assess the significance of the fixed effects (i.e., sampling times, distance to green space, distance to ground level, water regime, soil depth, planter area, establishment age, plant cover and plant species richness)**.

**Variables**	**numDF**	**denDF**	***F*-value**	***p*-value**
Plant species richness	1	29	6.04	0.02
Plant cover	1	230	675.41	<1 × 10^−4^
Establishment age	1	1	36.58	0.10
Area	1	1	97.28	0.06
Soil depth	1	29	10.11	3.5 × 10^−3^
Watering regime	1	29	27.64	<1 × 10^−4^
Distance to ground level	1	230	0.90	0.34
Distance to nearest green space	1	1	0.004	0.96
Sampling time	1	230	69.02	<1 × 10^−4^
Plant species richness: depth	1	27	8.75	6.4 × 10^−3^
Plant species richness: water	1	27	0.96	0.34
Plant species richness: sampling time	1	229	118.11	<1 × 10^−4^
Depth: water	1	27	1.06	0.31
Depth: sampling time	1	229	119.70	<1 × 10^−4^
Water: sampling time	1	229	330.32	<1 × 10^−4^

All arthropods sampled in our study were classified as either winged or wingless to provide an indication of arthropod motility and therefore colonization of urban plantings. We found both winged [*F*_(1, 29)_ = 6.09, *p* < 0.02; Table S1, Figure S2], and wingless [*F*_(1, 29)_ = 3.90, *p* < 0.05; Table S1, Figure S2] arthropod species richness positively correlated with plant species richness suggesting that arthropod community richness were not dependent purely on flight capability, and may therefore have a different origin.

## Discussion

Arthropod richness and plant productivity increased with plant richness and increased resource availability, showing that urban plantings can provide additional ecological values. Plant management practices were found to strongly influence the overall productivity of the plantings including light and water availability. Surprisingly, we found that neither planting age, nor planter distance from the ground were important predictors of arthropod richness or productivity. Overall, our findings are a promising result for the design and maintenance of urban plantings as we found a few easily managed characteristics can improve the productivity of urban plantings, i.e., the number of plant species, water, light availability, and soil properties.

Productivity was influenced by plant species richness, water, and climate in urban plantings. Ecological theory predicts that plant diversity can enhance productivity (e.g., plant biomass and overall energy flow), which in turn is linked to a wide range of ecosystem services, such as the assimilation of carbon (Waide et al., 1999). In our study, we found that plant diversity and plant cover had a significant positive relationship, but we also found that water regimes (and season) had a significant positive relationship with plant cover. The plantings with the highest species richness also received the highest levels of irrigation; therefore, water treatments are likely confounded with the plant diversity fixed effects. Surprisingly, other factors controlled by management practices such as establishment age, planter area (total area), soil depth, and PAR, did not have a significant relationship with plant cover.

Plant CO_2_ assimilation rates were influenced by PAR in urban plantings. Plant photosynthesis limits the amount of carbon that can be created and stored within ecosystems (Dias et al., 2010); and studies have found a positive relationship between plant diversity and carbon assimilation in natural systems (Conti and Díaz, 2013). Only two studies have examined green roof carbon sequestration potential, and although their findings suggest that greater plant biomass, and more complex plant communities increase CO_2_ uptake, the impact of plant diversity of this important service remains unknown (Getter et al., 2009; Whittinghill et al., 2014).

Contrary to previous studies, we found that neither plant species richness, nor plant cover have significant relationships with CO_2_ assimilation rates. Instead, and not surprisingly PAR was found to significantly influence CO_2_ assimilation rate, suggesting that other key requirements of plant productivity such as planter characteristics, and sampling times were not limiting factors.

### Soil CO_2_ efflux is influenced by soil temperature in urban plantings

Soil respiration is driven by autotrophic and heterotrophic respiration (Daily, 1997). The richness, amount of plant litter, and root exudates can influence soil respiration by altering soil chemistry. We found no significant relationship between either plant species richness and soil CO_2_ efflux, or plant cover and soil CO_2_ efflux; instead soil temperature was found to influence soil CO_2_ efflux.

Our result suggests that soil respiration within urban plantings may not be limited by either the amount, or the variety of dead plant material and plant root exudates. Instead, urban planting soil respiration may be due to the amount of soil organic matter derived from the pre-installed mulch and soils, although we did not test this here. If this is the case, plant diversity and productivity may not matter to soil respiration within urban plantings because the availability of soil organic matter is not a limiting factor. However, the plantings we investigated were between 1 and 6 years in age, so it is possible that plant diversity, and productivity may matter more in older plantings.

In our study, soil temperature was found to be positively correlated with soil CO_2_ efflux. Soil temperature is a critical limiting factor of soil organisms, especially microbes, which are the major contributors of all soil respiration (Yiqi and Zhou, 2010). Surprisingly, other environmental variables known to influence soil respiration such as age or water (Curiel Yuste et al., 2007), did not appear to have an influence. Landscapes accumulate more leaf litter, microbes and root biomass as they age (Matamala et al., 2008), yet age may not have been important as the plantings in our study were relatively young, ranging from 1 to 6 years, and this may be limiting soil forming processes (Schrader and Böning, 2006; Pavao-Zuckerman, 2008). Irrigation can either assist or inhibit the rate of plant litter decomposition by microbes depending on its availability (Curiel Yuste et al., 2007). In our study, irrigation was not significantly correlated with soil CO_2_ efflux.

Arthropod richness was influenced by plant species richness, water, and climate in urban plantings. Countless ecological studies of natural habitat have found plant richness, both amount and quality, influence arthropod richness (Siemann et al., 1998; Haddad et al., 2009). In city buildings, we also found both plant richness positively correlated with total arthropod richness on urban plantings, with plant richness having the stronger relationship. This result supports other studies that found a positive correlation between plant richness and invertebrate diversity in urban areas (Smith et al., 2006; Nielsen et al., 2014).

Even in a subtropical climate, season was found to influence total arthropod species richness, and the arthropod species richness of the three sampling methods. This finding is not surprising given that arthropods are more active due to the higher temperatures associated with seasonal change. The soil depth and the watering regime used by each respective building manager were correlated with arthropod species richness. Water is an essential resource requirement of many arthropod species, and was also found to be positively correlated with plant cover (Verhoef and Selm, 1983; Rumble and Gange, 2013). The positive correlation between soil depth and total arthropod richness is likely explained by more soil containing more resources, and niche space for arthropods (Byrne, 2007).

A fundamental understanding of how arthropods colonize urban plantings is needed if urban plantings are important as habitats for arthropods. Horizontal distance from the building site to the nearest green space was found in our study to have no influence on arthropod species richness or abundance, regardless of sampling method or motility classification. Some green roof studies have shown that the proximity of surrounding green space habitats has a positive effect on urban arthropod communities (Penone et al., 2012; Vergnes et al., 2012); however, other studies have shown surrounding habitats has no effect on arthropod richness at all (Schindler et al., 2011; Madre et al., 2013).

Urban plantings with diverse plant species may be able to support diverse arthropod communities, even if the plantings themselves are small and isolated from other habitats. Our study found that vertical distance from the plantings to the ground level had no influence on any measure of arthropod species richness or abundance. This provides a possible explanation as to why both, higher winged and wingless arthropod richness, were found in plantings with higher plant species richness, and supports the findings of Macivor and Lundholm (2011), who found a similar result in a comparative arthropod study between green roofs and adjacent level-ground habitats.

The species-area relationship is considered one of the strongest general theories in ecology (Huston, 1979), as it consistently holds across ecosystems. Although the sizes of urban plantings in our study were highly variable, ranging from 1 to 128 m^2^, area was only found to influence wingless arthropod species richness, and sticky trap arthropod richness. The sticky trap method was the only method used that is active−attracting arthropods; therefore, dispersal ability may again be a key explanation.

## Conclusions

Overall we found that the application of just a few easily managed characteristics of urban plantings such as plant species richness, access to light, water levels, and soil temperature can make a difference to the overall productivity of the plants and the arthropod diversity attracted. We measured pre-existing urban plantings that were managed differently, which was both a benefit and limitation of our study particularly since essential conditions for increased plant productivity such as water availability were highest for the plantings that had the highest species richness (although richness varied in the multiple plantings in each building). The benefit of measuring established urban plantings was that we were able to capture the natural variability between buildings and climatic conditions and therefore, were able to quantify more realistic trends than in a controlled experiment. Despite some confounding conditions, we found evidence that plant species richness and resource availability were strong drivers of arthropod richness and plant productivity.

### Conflict of interest statement

The authors declare that the research was conducted in the absence of any commercial or financial relationships that could be construed as a potential conflict of interest.
