# Supplementary material for: Diverse urban plantings managed with sufficient resource availability can increase plant productivity and arthropod diversity
Source: Front Plant Sci. 2014 Oct 30;5:517. doi: 10.3389/fpls.2014.00517 (PMC4214220; doi:10.3389/fpls.2014.00517)
Supplement: Supplementary file 1 [file DataSheet1.DOCX]

**Supplementary Material:**

**Diverse urban plantings managed with sufficient resource availability can increase plant productivity and arthropod diversity**

**Supplementary tables and figures**

**Table S1.** Sampling schedule for each site and sub-site.

| Site | Building level | Sub site | Sampling  Date (sampling time 1) | Sampling Date (sampling time 2) | Planter  area (m^2^) | Plant Species  Richness | Plant  Cover (%)  (Sampling time (1,2)) | Plant  Leaf CO_2_  Assimilation samples | Plant visual inspections | Number of Sticky Traps/  Soil cores | Soil CO_2_ respiration samples |
| --- | --- | --- | --- | --- | --- | --- | --- | --- | --- | --- | --- |
| One | Level2 | One2B | 24/07/2013 | 27/08/2013 | 5 | 3 | 76, 76 | 8 | 8 | 1 | 1 |
|  |  | One2C | 24/07/2013 | 27/08/2013 | 2 | 2 | 46, 45 | 4 | 4 | 1 | 1 |
|  |  | One2D | 24/07/2013 | 27/08/2013 | 8 | 7 | 63, 64 | 8 | 8 | 1 | 1 |
|  |  | One2E | 30/07/2013 | 05/09/2013 | 15 | 12 | 42, 42 | 8 | 8 | 1 | 1 |
|  |  | One2F | 30/07/2013 | 05/09/2013 | 1 | 1 | 20, 20 | 2 | 2 | 1 | 1 |
|  |  | One2G | 30/07/2013 | 05/09/2013 | 4 | 4 | 93, 93 | 8 | 8 | 1 | 1 |
|  | Level3 | One3A | 18/07/2013 | 21/08/2013 | <1 | 1 | 30, 30 | 2 | 2 | 1 | 1 |
|  |  | One3B | 18/07/2013 | 21/08/2013 | <1 | 2 | 10, 10 | 4 | 4 | 1 | 1 |
|  | Level6 | One6A | 18/07/2013 | 21/08/2013 | <1 | 2 | 65, 66 | 2 | 2 | 1 | 1 |
|  |  | One6B | 18/07/2013 | 21/08/2013 | <1 | 3 | 45, 47 | 2 | 2 | 1 | 1 |
|  | Level9 | One9A | 03/07/2013 | 21/08/2013 | <1 | 2 | 99, 100 | 2 | 2 | 1 | 1 |
|  |  | One9B | 03/07/2013 | 21/08/2013 | <1 | 2 | 100, 102 | 2 | 2 | 1 | 1 |
| Two | CP | TwoCPA | 01/08/2013 | 03/09/2013 | 35 | 5 | 80, 82 | 8 | 8 | 4 | 4 |
|  |  | TwoCPB | 01/08/2013 | 03/09/2013 | 40 | 6 | 80, 81 | 8 | 8 | 4 | 4 |
|  | Level1 | Two1A | 23/07/2013 | 30/08/2013 | 10 | 5 | 73, 74 | 8 | 8 | 1 | 1 |
|  |  | Two1B | 31/07/2013 | 30/08/2013 | 85 | 6 | 37, 37 | 8 | 8 | 8 | 8 |
|  |  | Two1C | 31/07/2013 | 30/08/2013 | 1 | 3 | 45, 46 | 4 | 4 | 1 | 1 |
|  | Level3 | Two3A | 17/07/2013 | 26/08/2013 | 1 | 4 | 35,35 | 8 | 8 | 1 | 1 |
|  |  | Two3B | 17/07/2013 | 26/08/2013 | 1 | 5 | 95, 95 | 8 | 8 | 1 | 1 |
|  |  | Two3G | 17/07/2013 | 26/08/2013 | 10 | 5 | 80, 82 | 8 | 8 | 2 | 2 |
|  | Level4 | Two4G | 12/07/2013 | 19/08/2013 | 10 | 5 | 90,93 | 8 | 8 | 2 | 2 |
|  |  | Two4H | 05/07/2013 | 19/08/2013 | 25 | 5 | 75, 77 | 8 | 8 | 2 | 2 |
|  | Level5 | Two5A | 12/07/2013 | 26/08/2013 | 1 | 4 | 80, 84 | 8 | 8 | 1 | 1 |
|  |  | Two5B | 12/07/2013 | 26/08/2013 | 1 | 5 | 48, 50 | 8 | 8 | 1 | 1 |
|  |  | Two6G | 01/07/2013 | 19/08/2013 | 10 | 3 | 47, 48 | 8 | 8 | 2 | 2 |
|  | Level7 | Two7A | 25/06/2013 | 19/08/2013 | 1 | 5 | 85, 86 | 8 | 8 | 1 | 1 |
|  |  | Two7B | 25/06/2013 | 19/08/2013 | 1 | 6 | 95, 97 | 8 | 8 | 1 | 1 |
| Three | Level4 | Three4A | 22/06/2013 | 07/09/2013 | 128 | 15 | 177.5, 184 | 8 | 8 | 12 | 12 |
|  |  | Three4B | 30/06/2013 | 03/08/2013 | 82 | 10 | 124, 128 | 8 | 8 | 8 | 8 |
|  |  | Three4C | 07/07/2013 | 12/08/2013 | 40 | 11 | 150, 160 | 8 | 8 | 4 | 4 |
|  | Level5 | Three5A | 28/07/2013 | 09/09/2013 | 60 | 9 | 179, 183 | 8 | 8 | 6 | 6 |
|  | Level6 | Three6A | 19/06/2013 | 06/09/2013 | 78 | 9 | 118, 134 | 8 | 8 | 20 | 20 |
|  |  | Three6B | 25/07/2013 | 06/09/2013 | 84 | 9 | 117, 128 | 8 | 8 | 20 | 20 |

**Table S2.** List of plant species on urban plantings

| Location | Plants |
| --- | --- |
| Building one | \| *Adiantum hispidulum* \| \| --- \| \| *Anthurium spp.* \| \| *Asplenium australasicum* \| \| *Bambusa textilis* \| \| *Calathea insignis* \| \| *Calathea sebrina* \| \| *Calathea spp.* \| \| *Clivia miniata* \| \| *Codiaeum variegatum* \| \| *Cordyline fruiticosa* \| \| *Epipiremnum aureum* \| \| *Neoregelia spp.* \| \| *Philodendron xanadu* \| \| *Sanseveria spp.* \| \| *Schefflera arboricola* \| \| *Solenostemon spp.* \| \| *Spathiphyllum spp.* \| \| *Trachelospermum jasminoides* \| \| *Tradescantia spathacea* \| \| *Unknown vine* \| |
| Building two | \| *Adiantum hispidulum* \| \| --- \| \| *Aechmea spp.* \| \| *Agathis robusta* \| \| *Alpinia zerumbet* \| \| *Alternanthera dentata* \| \| *Aspidistra elatior* \| \| *Bambusa textilis* \| \| *Boganvillea spp.* \| \| *Calathea insignis* \| \| *Calathea spp.* \| \| *Cissus antartica* \| \| *Citrus hystrix* \| \| *Citrus myersil* \| \| *Codiaeum variegatum* \| \| *Cordyline fruiticosa* \| \| *Crassula ovata* \| \| *Dieffenbachia seguine* \| \| *Hoya australis* \| \| *Lavandula stoechas* \| \| *Livistona australis* \| \| *Neoregelia carolinae* \| \| *Olea europaea* \| \| *Origanum majorana* \| \| *Passiflora spp.* \| \| *pyroslegia venusta* \| \| *Rhapis excelsa* \| \| *Rosmarinus officinalis* \| \| *Sanseveria spp.* \| \| *Thymus vulgaris* \| \| *Trachelospermum jasminoides* \| \| *Tradescantia spathacea* \| \| *Zamia furfuracae* \| |
| Building three | \| *Allocasia brisbanesis* \| \| --- \| \| *Alpinia spp.* \| \| *Angiopteris evecta* \| \| *Asplenium australasicum* \| \| *Atractocarpus randia* \| \| *Cissus antarctica* \| \| *Clivia miniata* \| \| *Cordyline petiolaris* \| \| *Cyathea cooperi* \| \| *Dianella caerula* \| \| *Doryanthes excelsa* \| \| *Grevillea spp.1* \| \| *Grevillea spp.2* \| \| *Lepidozamia peroffskyana* \| \| *Livistona australis* \| \| *Lomandra longifolia* \| \| *Monster deliciossa* \| \| *Syngonium podophyllum* \| \| *Thunbergia mysorensis* \| \| *Unknown bush* \| \| *Unknown heart vine* \| \| *Unknown spiny leaf* \| |

**Table S3.** Summary of arthropod species richness and abundance [richness(abundance)] for each urban planting site for each sampling time (ST), for each sampling method (all, visual, sticky and soil), and for dispersal capability (winged, wingless).

| Site | Order | All arthropods | | Visual arthropods | | Sticky arthropods | | Soil arthropods | | Winged arthropods | | Wingless arthropods | |
| --- | --- | --- | --- | --- | --- | --- | --- | --- | --- | --- | --- | --- | --- |
|  |  | ST1 | ST2 | ST1 | ST2 | ST1 | ST2 | ST1 | ST2 | ST1 | ST2 | ST1 | ST2 |
| One | Amphipoda | 1(73) | 1(74) | 0(0) | 0(0) | 0(0) | 0(0) | 1(73) | 1(74) | 0(0) | 0(0) | 1(73) | 1(74) |
|  | Aranae | 0(0) | 1(1) | 0(0) | 1(1) | 0(0) | 0(0) | 0(0) | 0(0) | 0(0) | 0(0) | 0(0) | 1(1) |
|  | Blattodea | 1(1) | 0(0) | 0(0) | 0(0) | 1(1) | 0(0) | 0(0) | 0(0) | 1(1) | 0(0) | 0(0) | 0(0) |
|  | Coleoptera | 0(0) | 1(3) | 0(0) | 0(0) | 0(0) | 1(2) | 0(0) | 1(1) | 0(0) | 1(3) | 0(0) | 0(0) |
|  | Diptera | 11(67) | 9(55) | 0(0) | 0(0) | 10(63) | 9(53) | 1(4) | 0(0) | 11(67) | 9(53) | 0(0) | 0(0) |
|  | Entomobryomorpha | 0(0) | 2(5) | 0(0) | 0(0) | 0(0) | 0(0) | 0(0) | 2(5) | 0(0) | 0(0) | 0(0) | 2(5) |
|  | Geophilomorpha | 0(0) | 2(10) | 0(0) | 0(0) | 0(0) | 0(0) | 0(0) | 1(10) | 0(0) | 0(0) | 0(0) | 2(10) |
|  | Hemiptera | 7(419) | 6(1140) | 5(408) | 6(1140) | 1(1) | 0(0) | 0(0) | 0(0) | 1(1) | 1(1044) | 5(418) | 5(96) |
|  | Hymenoptera | 1(8) | 3(57) | 1(1) | 2(50) | 1(7) | 2(7) | 0(0) | 0(0) | 0(0) | 1(1) | 1(8) | 2(56) |
|  | Isopoda | 1(4) | 1(13) | 0(0) | 0(0) | 0(0) | 0(0) | 1(4) | 1(13) | 0(0) | 0(0) | 1(4) | 1(13) |
|  | Polydesmida | 1(2) | 1(3) | 0(0) | 0(0) | 0(0) | 0(0) | 1(2) | 1(3) | 0(0) | 0(0) | 1(2) | 1(3) |
|  | Scolopendromorpha | 0(0) | 1(4) | 0(0) | 0(0) | 0(0) | 0(0) | 0(0) | 1(4) | 0(0) | 0(0) | 0(0) | 1(4) |
|  | Trombidiformes | 1(2) | 1(1) | 0(0) | 0(0) | 0(0) | 0(0) | 1(2) | 1(1) | 0(0) | 0(0) | 1(2) | 1(1) |
| Two | Amphipoda | 0(0) | 1(14) | 0(0) | 0(0) | 0(0) | 0(0) | 0(0) | 1(14) | 0(0) | 0(0) | 0(0) | 1(14) |
|  | Aranae | 2(4) | 2(2) | 0(0) | 2(2) | 2(4) | 0(0) | 0(0) | 0(0) | 0(0) | 0(0) | 2(4) | 2(2) |
|  | Blattodea | 0(0) | 1(8) | 0(0) | 0(0) | 0(0) | 0(0) | 0(0) | 1(8) | 0(0) | 0(0) | 0(0) | 1(8) |
|  | Coleoptera | 9(82) | 4(26) | 0(0) | 2(2) | 8(72) | 3(9) | 1(10) | 3(14) | 9(82) | 4(26) | 0(0) | 0(0) |
|  | Diptera | 20(420) | 14(410) | 0(0) | 3(3) | 18(419) | 9(387) | 1(1) | 2(20) | 20(420) | 13(410) | 0(0) | 0(0) |
|  | Entomobryomorpha | 1(21) | 3(64) | 0(0) | 0(0) | 0(0) | 0(0) | 1(21) | 3(60) | 0(0) | 0(0) | 1(21) | 3(60) |
|  | Geophilomorpha | 0(0) | 1(9) | 0(0) | 0(0) | 0(0) | 0(0) | 0(0) | 1(9) | 0(0) | 0(0) | 0(0) | 1(9) |
|  | Hemiptera | 14(2255) | 11(2945) | 10(1922) | 9(2490) | 5(333) | 3(535) | 0(0) | 0(0) | 7(471) | 3(564) | 8(1784) | 8(2381) |
|  | Hymenoptera | 9(64) | 6(140) | 0(0) | 3(124) | 8(61) | 2(5) | 2(3) | 2(11) | 5(8) | 2(5) | 4(56) | 4(135) |
|  | Isopoda | 1(15) | 2(19) | 0(0) | 0(0) | 0(0) | 0(0) | 1(15) | 2(19) | 0(0) | 0(0) | 1(15) | 2(19) |
|  | Orthoptera | 1(1) | 2(2) | 0(0) | 2(2) | 1(1) | 0(0) | 0(0) | 0(0) | 1(1) | 2(2) | 0(0) | 0(0) |
|  | Polydesmida | 0(0) | 1(3) | 0(0) | 0(0) | 0(0) | 0(0) | 0(0) | 1(3) | 0(0) | 0(0) | 0(0) | 1(3) |
|  | Psocoptera | 2(35) | 1(40) | 0(0) | 0(0) | 2(35) | 1(40) | 0(0) | 0(0) | 2(35) | 1(40) | 0(0) | 0(0) |
|  | Scolopendromorpha | 1(2) | 2(17) | 0(0) | 0(0) | 0(0) | 0(0) | 1(2) | 2(17) | 0(0) | 0(0) | 1(2) | 2(17) |
|  | Thysanoptera | 0(0) | 1(141) | 0(0) | 1(141) | 0(0) | 0(0) | 0(0) | 0(0) | 0(0) | 1(141) | 0(0) | 0(0) |
|  | Trombidiformes | 2(15) | 1(41) | 0(0) | 0(0) | 0(0) | 0(0) | 2(15) | 1(41) | 0(0) | 0(0) | 2(15) | 1(41) |
| Three | Amphipoda | 2(12) | 1(55) | 0(0) | 0(0) | 0(0) | 0(0) | 2(12) | 1(55) | 0(0) | 0(0) | 2(12) | 1(55) |
|  | Araneae | 3(5) | 3(19) | 0(0) | 1(1) | 3(5) | 3(18) | 0(0) | 0(0) | 0(0) | 0(0) | 3(5) | 3(19) |
|  | Blattodea | 1(14) | 3(14) | 0(0) | 0(0) | 0(0) | 3(14) | 1(14) | 2(12) | 0(0) | 1(2) | 1(14) | 2(11) |
|  | Coleoptera | 7(222) | 9(491) | 0(0) | 4(11) | 6(176) | 6(408) | 2(44) | 3(72) | 8(220) | 9(491) | 0(0) | 0(0) |
|  | Dermaptera | 1(1) | 1(1) | 0(0) | 0(0) | 0(0) | 0(0 | 1(1) | 1(1) | 0(0) | 0(0) | 1(1) | 1(1) |
|  | Diptera | 19(958) | 19(1250) | 0(0) | 6(11) | 18(951) | 18(1239) | 1(7) | 0(0) | 19(958) | 19(1250) | 0(0) | 0(0) |
|  | Entomobryomorpha | 2(40) | 4(160) | 0(0) | 0(0) | 0(0) | 0(0) | 2(40) | 4(160) | 0(0) | 0(0) | 2(40) | 4(160) |
|  | Geophilomorpha | 1(3) | 1(7) | 0(0) | 0(0) | 0(0) | 0(0) | 1(3) | 1(7) | 0(0) | 0(0) | 1(3) | 1(7) |
|  | Hemiptera | 19(903) | 18(678) | 16(825) | 16(610) | 5(78) | 7(52) | 0(0) | 0(0) | 11(471) | 9(169) | 12(432) | 9(509) |
|  | Hymenoptera | 8(31) | 13(254) | 0(0) | 3(47) | 7(17) | 10(29) | 2(14) | 5(174) | 3(10) | 8(29) | 5(21) | 5(225) |
|  | Isopoda | 2(27) | 4(80) | 0(0) | 0(0) | 0(0) | 0(0) | 2(27) | 4(80) | 0(0) | 0(0) | 2(27) | 4(80) |
|  | Lepidoptera | 1(4) | 1(9) | 1(3) | 0(0) | 1(1) | 4(6) | 0(0) | 1(3) | 1(1) | 1(9) | 1(3) | 0(0) |
|  | Neuroptera | 1(2) | 1(11) | 0(0) | 0(0) | 1(2) | 1(11) | 0(0) | 0(0) | 1(2) | 1(11) | 0(0) | 0(0) |
|  | Orthoptera | 0(0) | 3(4) | 0(0) | 2(2) | 0(0) | 0(0) | 0(0) | 1(2) | 0(0) | 1(1) | 0(0) | 2(3) |
|  | Pseudoscorpionida | 1(26) | 1(6) | 0(0) | 0(0) | 0(0) | 0(0) | 1(26) | 1(6) | 0(0) | 0(0) | 1(26) | 1(6) |
|  | Psocoptera | 1(81) | 2(58) | 0(0) | 0(0) | 1(81) | 2(58) | 0(0) | 0(0) | 1(81) | 2(58) | 0(0) | 0(0) |
|  | Scolopendromorpha | 2(12) | 2(19) | 0(0) | 0(0) | 0(0) | 0(0) | 2(12) | 2(19) | 0(0) | 0(0) | 2(12) | 2(19) |
|  | Thysanoptera | 1(234) | 2(1515) | 1(121) | 2(1346) | 1(113) | 1(169) | 0(0) | 0(0) | 1(234) | 2(1515) | 0(0) | 0(0) |
|  | Trombidiformes | 2(125) | 2(135) | 0(0) | 0(0) | 0(0) | 0(0) | 2(125) | 2(135) | 0(0) | 0(0) | 2(125) | 2(135) |

**Table S1.** Results for each ANOVA conducted to assess the significance of the cloud cover (eighths), humidity (%), precipitation (mm), and air temperature (°C) on sampling times.

| Response variables | df | F-value | *p*-value |
| --- | --- | --- | --- |
| Cloud cover | 1-28 | 6.15 | 0.02 |
| Humidity | 1-28 | 10.41 | <0.01 |
| Precipitation | 1-28 | 2.07 | <0.01 |
| Air temperature | 1-28 | 14.58 | 0.16 |

**Table S2.** Results from an ANOVA conducted to assess the significance of the fixed effects (i.e. sampling times, distance to green space, distance to ground level, watering regime, soil depth, area, establishment age, plant coverage and plant species richness)on a linear mixed effects model for winged arthropod species richness.

| Variables | numDF | denDF | F-value | *p*-value |
| --- | --- | --- | --- | --- |
| Plant species richness | 1 | 29 | 6.09 | 0.02 |
| Plant cover | 1 | 230 | 3.48 | 0.06 |
| Age | 1 | 1 | 31.89 | 0.11 |
| Area | 1 | 1 | 1.58 | 0.43 |
| Soil depth | 1 | 29 | 4.22 | 0.05 |
| Watering regime | 1 | 29 | 7.83 | 9x10^-3^ |
| Distance ground | 1 | 230 | 0.044 | 0.83 |
| Distance green space | 1 | 1 | 0.05 | 0.86 |
| Sampling time | 1 | 230 | 297.60 | <0.01 |
| Plant species richness: sampling time | 1 | 229 | 32.05 | <0.01 |

**Table S3.** Results from an ANOVA conducted to assess the significance of the fixed effects (i.e. sampling times, distance to green space, distance to ground level, watering regime, soil depth, area, establishment age, plant coverage and plant species richness)on a linear mixed effects model for wingless arthropod species richness

| Variables | numDF | denDF | F-value | *p*-value |
| --- | --- | --- | --- | --- |
| Plant species richness | 1 | 29 | 3.90 | 0.05 |
| Coverage | 1 | 230 | 38.27 | <0.01 |
| Age | 1 | 1 | 16.30 | 0.16 |
| Area | 1 | 1 | 244.59 | 0.04 |
| Soil depth | 1 | 29 | 7.35 | 0.01 |
| Watering regime | 1 | 29 | 40.22 | <1x10^-4^ |
| Distance ground | 1 | 230 | 1.71 | 0.19 |
| Distance green space | 1 | 1 | <0.01 | 0.99 |
| Sampling time | 1 | 230 | 315.75 | <0.01 |
| Plant species richness: age | 1 | 28 | 0.76 | 0.39 |
| Plant species richness: depth | 1 | 27 | 3.29 | 0.08 |
| Plant species richness: sampling time | 1 | 229 | 157.91 | <0.01 |


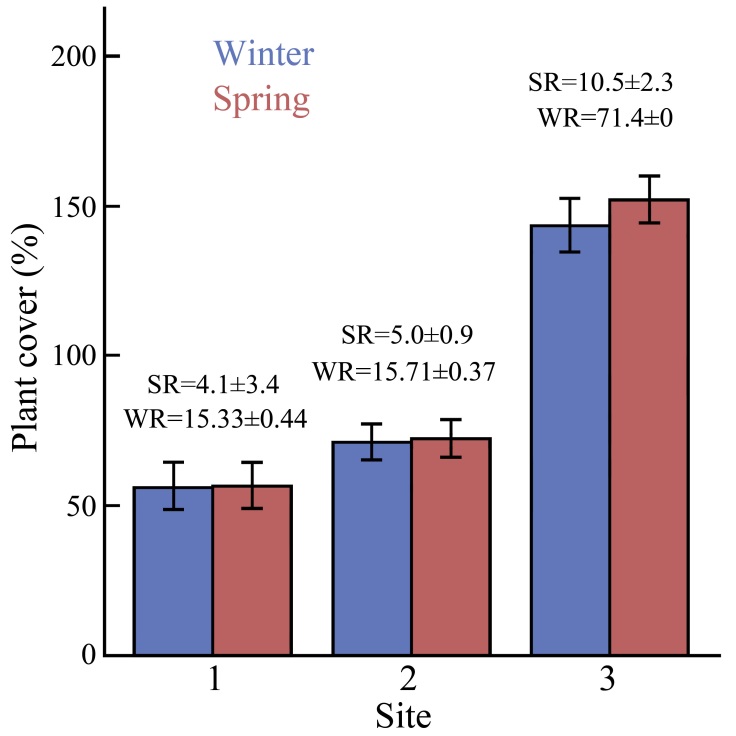


**Figure S1:** Comparison of plant cover (%) from different building sites depending on plant species richness (SR=mean SR per building) and watering regime (WR=mean water regime per building; Litres/1m^2^/week), and how this relationship varies between sampling times. Error bars indicate 95% confidence intervals


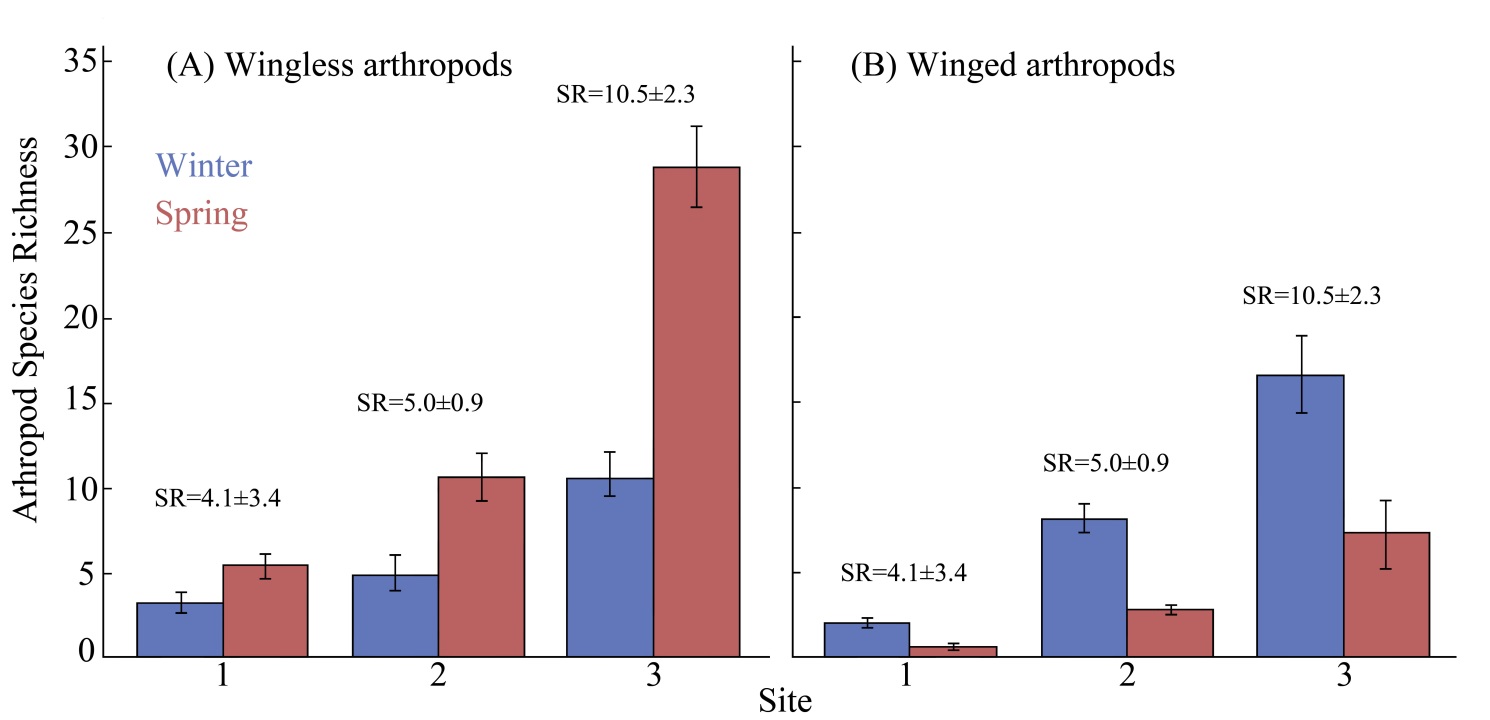


**Figure S2:** Comparison of winged and wingless arthropod species richness at different sites depending on plant species richness (SR), and how this relationship varies between sampling times. Error bars indicate 95% confidence intervals.
